# Integrated scheduling of distributed production and distribution in group manufacturing with uncertain travel time

**DOI:** 10.1007/s40747-022-00875-7

**Published:** 2022-10-15

**Authors:** Jun Guo, Wenjun Liu, Zhao Peng, Baigang Du

**Affiliations:** 1 School of Mechanical and Electronic Engineering, Wuhan University of Technology, Wuhan, 430070 China; 2 Hubei Digital Manufacturing Key Laboratory, Wuhan, 430070 China

**Keywords:** Integrated production and distribution scheduling, Group manufacturing, Uncertain travel time, Improved genetic algorithm, Tabu search

## Abstract

This paper presents a novel integrated distributed production and distribution scheduling problem in group manufacturing with uncertain travel time (IDPDSP-GM-UTT), in which products are firstly produced in several distributed hybrid flow shops and then delivered to several retailers in batches. The proposed model considers both geographical dispersion of multi-factories and variable travel time between factories and retailers caused by time-varying dynamics of road network, which describes the production environment more authentic. Additionally, a mathematical model is developed to find the optimal quantity of raw material, delivery plan, and punishment of earliness and tardiness with the objective of minimizing total costs. Then, an improved genetic algorithm with two-stage heuristic mutation scheduling strategy and tabu search for local optimization (GA-2HMS&TS) is designed to solve the proposed model. To verify the performances of the proposed method, several experiments by adopting test experimental examples with different scales are performed. The computational results exhibit that the GA-2HMS&TS not only significantly reduces the total cost of production and distribution, but also outperforms all of its rivals. In addition, the robustness of the proposed models is also analyzed with regard to the different road conditions.

## Introduction

Production and distribution are two crucial activities in group manufacturing [1], and their scheduled planning are separately treated in most cases [2]. However, this decentralized approach leads to inefficiency, due to the complex organizational structure and untimely information transmission in manufacturing group. Thus, they need to be considered together to improve the production efficiency and reduce the operation cost of the manufacturing group [3]. Moreover, considering the difficulty in coordinating behavior of different subsidiaries, integrated production and distribution scheduling problem is one of the most important challenges facing by the group manufacturing.

As the economic globalization and demand diversification, the factories of manufacturing group are established with decentralized geographic location and independent operating agency [4]. What’s more, due to the similar or identical production process, manufacturing group usually adopts the hybrid flow-shop mode which can balance the utilization rate of machines, increase production capacity and improve the efficiency of production lines. And the distribution stage is always addressed as a batch distribution problem of multiple vehicles and multiple customers where the capacity of vehicles is limited. Thus, the integrated production and distribution scheduling problem in group manufacturing can be regarded as a combinatorial optimization problem. It needs to handle the distributed hybrid flow-shop scheduling problem (DHFSP) at the production stage [5, 6], and multi-vehicle multi-customer batch delivery scheduling problem at the distribution stage simultaneously.

Additionally, the current turbulent business circumstances and the ever-changing market in the context of COVID-19 present new challenges to decision makers. There are many uncertain factors in the process of distribution, such as bad traffic, bad weather and unpredictable impacts. This time-varying dynamic of road network usually leads to the inaccuracy of model, and then incurs unnecessary losses if these factors are ignored. The dynamic nature of the environment determines that the scheduling environment is not deterministic [7]. Only by adapting to uncertain factors can companies obtain better competitiveness and certification.

To solve the aforementioned problems, we propose a novel integrated distributed production and distribution scheduling problem in group manufacturing considering uncertain travel time (IDPDSP-GM-UTT) to minimize the total cost. The main contributions of this work can be summarized as follows:

- We investigate a joint distributed hybrid flow-shop production and batch distribution scheduling with uncertain travel time problem in group manufacturing, which is discussed in few works in literature although this mode is implemented in a broad range of actual manufacturing scenarios.
- To cope with the complexity of IDPDSP-GM-UTT, we propose an improved genetic algorithm with two-stage heuristic mutation scheduling strategy and tabu search. Two set of benchmark test examples are presented to illustrate the effectiveness of the proposed model and solution algorithm. Moreover, the experiments are extended by including additional analysis of robustness and influence of uncertain factors regarding the different road conditions.

The rest of this paper is structured as follows. “Literature reviews” shows the relevant literature on the investigated problem. “Problem formulation” describes the IDPDSP-GM-UTT and provides a mathematical model. “Proposed solution algorithm” describes an improved genetic algorithm with two-stage heuristic mutation scheduling strategy and tabu search for local optimization (GA-2HMS&TS) with its search operators. “Experimental results and discussions” presents the computational results and performance analysis. Finally, conclusions and expectations for the future research are given in “Conclusion”.

## Literature reviews

In recent years, many scholars devote themselves to the research of integrated production and distribution scheduling problem (IPDSP) for the sake of saving costs and improving competitiveness of enterprises [8]. Agnetis et al. [9] addressed an IPDSP for supply chain coordination, in which there was only one manufacturer and the order of products was pre-specified. Yağmur and Kesen [10] studied an IPDSP where a single manufacturer processed jobs and subsequently distributed by a single capacitated vehicle. Fu et al. [11] proposed an IPDSP for metal packaging industry considering the job splitting and delivery time windows, and they developed a two-phase iterative heuristic to solve it. Kergosien et al. [12] presented an IPDSP for a healthcare industry, and a heuristic based on benders decomposition was introduced to handle it. Cheng et al. [13] studied an IPDSP for manufacturers, in which jobs in a batch were processed together. Besides, some researchers discussed more details about the different production models, such as single-machine [14, 15], parallel-machine [16, 17], flow shop [18, 19], and job shop [20]. Although the above literatures consider the different production modes, they focus on single factory at the production stage.

In addition to the traditional consideration of transport constraints, Senoussi et al. [21] assumed that the distance between the supplier and the retailer was fixed, and they designed five heuristics based on genetic algorithms to solve production distribution problem. Ganji et al. [22] studied an integrated scheduling problem considering due-dates with production and distribution times. Wang et al. [23] addressed an IPDSP, where distribution stage aimed at solving a vehicle routing problem. And an improved MA was designed to handle it. Guo et al. [24] considered several transportation modes with different vehicle capacities and transportation times in the IPDSP. Vincenzo et al. [25] considered an IPDSP for non-perishable food and beverage industry. They introduced a metaheuristic algorithm to find approximate solutions. In the perishable goods industry, Devapriya et al. [26] proposed an IPDSP for perishable products with constrained fleet size and vehicles’ routes, and they developed evolutionary algorithms to solve it. Liu and Liu [27] presented an IPDSP with vehicles capacity constraints for perishable products, and an improved large neighborhood search algorithm was designed to handle it. Liu et al. [28] addressed an integrated production‑inventory‑routing of blood in a supply chain network, which consisted of a single supplier and a group of blood centers. And a heuristic solution algorithm was developed to deal with it. Wei et al. [29] researched on production-delivery-inventory strategies considering the inventory and delivery sustainability of perishable products.

By summarizing the relevant researches on IPDSP, we can find that most of existing literature consider scheduling single factory at the production stage. There are few studies give attention on the integrated distributed production and distribution scheduling problems (IDPDSP) although they actually exist in a wide range of modern manufacturing scenarios. Marandi and Ghomi [6] introduced an integrated multi-factory production and distribution scheduling problem with the objective of minimizing the sum of tardiness cost and transportation cost. In their model, a number of factories were joined together in a network configuration. And an improved imperialist competitive algorithm was developed to solve it. Badhotiya et al. [30] proposed a fuzzy multi-objective mixed integer programming model considering multi-site manufacturing environment. They used piecewise linear membership function to represent three fuzzy objectives of total cost, delivery time, and backorder level. Hou et al. [31] studied an integrated model with the consideration of time windows of limited vehicles in a multi-factory manufacturing system. They established a mixed integer programming model to minimize total weighted earliness and tardiness and developed an enhanced brain storm optimization algorithm to deal with it. Qin et al. [32] addressed an IDPDSP in distributed hybrid flow shop environment and multiple transportation modes to minimize the sum of earliness, tardiness and delivery costs. They introduced an adaptive human-learning-based genetic algorithm to handle it. However, the above studies on IDPDSP do not take into account the time variability in the logistics distribution process. In the actual urban road network, the vehicle speed is always changing due to the influence of traffic management, traffic flow, traffic accidents, rush hours and other factors. If this factor is not taken into account, it may lead to excessive value loss caused by prolonged waiting time of distribution vehicles or delayed product delivery, which reduces the profitability and service level of group enterprises. Thus, this work describes a novel IDPDSP model considering uncertain travel time. A mixed integer programming model is formulated and an improved genetic algorithm with two-stage heuristic mutation scheduling strategy and tabu search for local optimization (GA-2HMS&TS) is designed to find the optimal solution of total cost. Then, experimental comparisons under different road conditions are also made to analyze the impact of uncertain factors on time window violation.

## Problem formulation

### Problem description

**Fig. 1:**
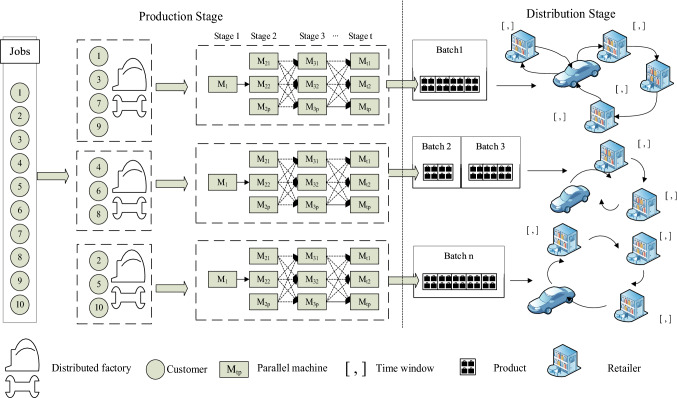
Integrated production and distribution in group manufacturing

The IDPDSP-GM-UTT is a new combinatorial optimization problem, which consists of two inter-dependent sub-problems, namely DHFSP in the production stage and batch delivery of finished products with capacity limited multi-vehicles and multi-customer in the distribution stage, as indicated in Fig. 1.

In the production stage, a set of *n* jobs are assigned to *f* production factories with different geographical locations for processing. Each of the factories is arranged as a hybrid flow shop, which has a set of *t* stages and *m*_*tp*_ parallel machines. A set of jobs are randomly assigned among factories. Each job has to go through a set of sequential stages in every factory. And at each stage, a set of parallel and identical machines are available to process the job. To be more consistent with the actual factory situation, it is assumed that there is only one machine in the first stage of each factory. In the distribution stage, it includes the batch division of jobs and assignment of delivery vehicles, which can be addressed as a traveling scheduling problem with multiple distribution points locating in different regions and customers with time window requirements. And the cost of single delivery is related only to the length of the delivery route and not to the number of jobs. Considering the geographically dispersed nature of the factories, the rules for dividing batches are based on the completion time of jobs. And jobs processed at the same factory will be divided into the same batch unless the number contained reaches the vehicle capacity limit.

To facilitate the modeling and solving of the IDPDSP-GM-UTT, some assumptions are as follows.

1. Assignment of jobs is random and any factory can process all the jobs.
2. The division of jobs batches is random in initialization.
3. There are enough parallel machines at any stages to satisfy scheduling needs except the first stage of workpiece.
4. The production capacity of each factory is consistent.
5. The finished products are distributed according to the divided batches;
6. Once a job is assigned to a definite factory, all its steps ought to be executed in this factory and interruption is not allowed;
7. One machine can only process one job at a time, and jump the queue is not allowed;
8. There are no buffer limits between stages;
9. The production preparation time, product packaging time, product loading time and product unloading time of mechanical products are not take offense in this article.

### Mathematical model

Considering the geographical dispersion of the factories and multi-vehicle delivery method adopted, this section proposes a mathematical model for IDPDSP-GM-UTT. The indexes, parameters and variables are defined as follows.

**Table:** 

Indexes	
*p*	Index of jobs, $1 \le p \le n$
*t*	Index of stages, $1 \le t \le T$
*f*	Index of factories, $1 \le f \le F$
*l*	Index of parallel machine
*v*	Index of vehicle, $1 \le v \le V$
*i,* *j*	Index of retailer, $1 \le i \le r,1 \le j \le r$
*h*	Index of batch distribution loop, $1 \le h \le V$
*b*	Index of batches, $1 \le b \le n$
Parameters	
*F*	The number of factories
*n*	The number of released jobs
*V*	The number of vehicles used in a single dispatch
*T*	The number of stages
$d_{f,i}^{{}}$	Distance from factory f to retailer *i*
$d_{i,j}^{{}}$	Distance from retailer *i* to retailer j
r	The number of retailers in a single dispatch
M	A sufficiently large number
$\tau_{v}^{{}}$	Capacity limits of vehicle *v*
$\left[ {\zeta_{{\text{p}}}^{{}} ,\xi_{{\text{p}}}^{{}} } \right]$	Customer's time window of job *p*
$\pi_{f}^{{\text{p}}}$	*j*th job of the job *p* sequence processed in factory *f*
$\upsilon (\pi_{f}^{{\text{p}}} ,t)$	Processing time of job *p* at stage *t*
$CP(\pi_{f}^{{\text{p}}} ,t)$	Unit processing cost of job *p* at stage t in factory *f*
$a(\pi_{f}^{{\text{p}}} ,b)$	Delivery time of job *p* in factory *f*
$VT_{{{\text{f}},p}}^{{}}$	Departure time of job *p* in factory *f*
${\text{TC}}_{{i,{\text{p}}_{{}} }}^{{}}$	Unit tardiness cost of job *p* for retailer *i*
${\text{EC}}_{{i,p_{{}} }}^{{}}$	Unit earliness cost of job *p* for retailer *i*
$CR_{{\text{p}}}^{{}}$	Raw material cost of job *p*
$\psi_{v}^{{}}$	Original cost of vehicle *v*
$T(\pi_{f}^{{\text{p}}} ,t)$	Production waiting time of job *p* in factory *f*
$t_{i,j}^{{}}$	Travel time from retailer *i* to retailer *j*
$\omega_{v}^{{}}$	variable transportation cost of vehicle *v*
$V_{p}$	Vehicle index of job *p*
Decision variables	
$A_{{{\text{ftlp}}_{1} {\text{p}}_{2} }}^{{}}$	1 if the processing sequence of job $p_{1}$ and job $p_{2}$ are adjacent on machine l at stage *t* in factory *f*; and 0 otherwise
$B(\pi_{f}^{{\text{p}}} ,{\text{b)}}$	1 if batch *b* contains job *p*; and 0 otherwise
$C(\pi_{f}^{{\text{p}}} {\text{,b}})$	1 if the products of job $\pi_{f}^{{\text{p}}}$ are manufactured in factory *f*; and 0 otherwise
$D(\pi_{f}^{{\text{p}}} ,i)$	1 if the products of job $\pi_{f}^{{\text{p}}}$ belong to retailer *i*; and 0 otherwise
$E_{{{\text{fp}}_{1} {\text{p}}_{2} }}^{{}}$	1 if job $p_{1}$ and job $p_{2}$ are in the same vehicle; and 0 otherwise
$\sigma (\pi_{f}^{{\text{p}}} ,t)_{{}}^{{}}$	Production finished time of job $\pi_{f}^{{\text{p}}}$ at stage *t* in factory *f*
$V_{{\text{b}}}^{{}}$	Delivery departure time of batch *b*

The objective function and constraints are as follows:

1 $$ MIN\alpha = \left\{ \begin{gathered} \sum\limits_{p = 1}^{P} {CR_{{\text{p}}}^{{}} } { + }\sum\limits_{p = 1}^{P} {\sum\limits_{f = 1}^{F} {\sum\limits_{t}^{T} {CP(\pi_{f}^{{\text{p}}} ,t)\upsilon (\pi_{f}^{{\text{p}}} ,t)} } } + \sum\limits_{v = 1}^{V} {\psi_{v}^{{}} } + \sum\limits_{p}^{P} {\sum\limits_{v = 1}^{V} {\sum\limits_{i = 1,j = 1}^{n} {\sum\limits_{b}^{B} {\omega_{v}^{{}} t_{i,j}^{{}} B(\pi_{f}^{{\text{p}}} ,{\text{b)}}} } } } \hfill \\ + \sum\limits_{p = 1}^{P} {\sum\limits_{i = 1}^{n} {\sum\limits_{{\text{f}}}^{F} {{\text{EC}}_{{i,p_{{}} }}^{{}} \times \max \{ 0,\zeta_{{\text{p}}}^{{}} - a_{{{\text{f}},p}}^{{}} \} } } } + \sum\limits_{p = 1}^{P} {\sum\limits_{i = 1}^{n} {\sum\limits_{{}}^{F} {{\text{TC}}_{{i,{\text{p}}_{{}} }}^{{}} \times \max \{ a_{{{\text{f}},p}}^{{}} - \xi_{{\text{p}}}^{{}} \} } } } \hfill \\ \end{gathered} \right\} $$

*s*.*t*

2 $$ \sigma (\pi_{f}^{{\text{p}}} ,{\text{t}}) \ge \upsilon (\pi_{f}^{{\text{p}}} ,t),\forall f,p,t $$

3 $$ \sigma (\pi_{f}^{{{\text{p}}_{2} }} ,1) \ge \sum\limits_{l = 1}^{{m_{1} }} {\sum\limits_{{j_{1} = 1}}^{{n_{i} }} {A_{{{\text{ftlp}}_{1} {\text{p}}_{2} }}^{{}} \sigma (\pi_{f}^{1} ,1)} } + \upsilon (\pi_{f}^{{{\text{p}}_{2} }} ,1),\forall f $$

4 $$ T(\pi_{f}^{{\text{p}}} ,t){ = 0,}\forall p,f,t = 1 $$

5 $$ \upsilon (\pi_{f}^{{\text{p}}} ,t) \le T(\pi_{f}^{{\text{p}}} ,t),\forall p,f,t $$

6 $$ V_{{\text{b}}}^{{}} = \sum\limits_{p = 1}^{f} {C(\pi_{f}^{{\text{p}}} {\text{,b}})\max \{ B(\pi_{f}^{{\text{p}}} ,{\text{b)}}\sigma (\pi_{f}^{{\text{p}}} ,t)_{{}}^{{}} \} } ,\forall f,{\text{b}},p $$

7 $$ \sigma (\pi_{f}^{{\text{p}}} ,t)_{{}}^{{}} \le VT_{{{\text{f}},p}}^{{}} ,\forall f,t,p $$

8 $$ \left[ {\zeta_{{\text{p}}}^{{}} \le a(\pi_{f}^{{\text{p}}} ,b) \le \xi_{{\text{p}}}^{{}} } \right],\forall i,v,p $$

9 $$ V_{{\text{b}}}^{{}} B(\pi_{f}^{{\text{p}}} ,{\text{b)}} + VT_{{{\text{f}},p}}^{{}} \le a(\pi_{f}^{{\text{p}}} ,b),\forall p,f,b $$

10 $$ a(\pi_{f}^{{\text{p}}} ,b){ = }\sum\limits_{i,j(i \ne j)}^{n} {E_{{{\text{fp}}_{1} {\text{p}}_{2} }}^{{}} t_{i,j}^{{}} } { + }V_{{\text{b}}}^{{}} B(\pi_{f}^{{\text{p}}} ,{\text{b)}},\forall i,p,v $$

11 $$ \sum\limits_{p \in P}^{{}} {B(\pi_{f}^{{\text{p}}} ,{\text{b)}}} = 1 $$

12 $$ B_{i,j}^{{}} \le E_{(i,j)v} ,\forall i,j,v(i \ne j) $$

13 $$ {1} \le \sum\limits_{{{\text{i}} = 1}}^{n} {E_{{{\text{fp}}_{1} {\text{p}}_{2} }}^{{}} } \le \tau_{v}^{{}} ,\forall f,p,v $$

14 $$ \sum\limits_{{{\text{i}} \in n}}^{{}} {D(\pi_{f}^{{\text{p}}} ,i)} = 1,\forall f,p,i $$

15 $$ \sum\limits_{p \in P}^{{}} {C(\pi_{f}^{{\text{p}}} {\text{,b}})} = 1,\forall f,p,b $$

16 $$ B(\pi_{f}^{{\text{p}}} ,{\text{b)}}VT_{{{\text{f}},p}}^{{}} > 0,\forall f,p,b $$

17 $$ \sigma (\pi_{f}^{{\text{p}}} ,t) > 0,V_{{\text{b}}}^{{}} > 0,\omega_{v}^{{}} > 0,\forall f,p,v,{\text{b}},t $$

where Eq. (1) represents that the objective is to minimize the total costs of raw material, production, vehicle, delivery, earliness, tardiness. Constraints (2) and (3) ensure that the finished time of jobs are correct. Constraints (4) guarantees that the production wait time of the first stage of job is zero. Constraints (5) represents the waiting time of job *p* is greater than or equal to the processing time of job *p*. Constraints (6) determines that the distribution of batch *b* start after all the jobs in batch *b* have been completed. Constraint (7) represents the production completion time of job *p* is less than or equal to the delivery departure time of the batch *b* which contains job *p*. Constraints (8) is the time window limit for job *p*. Constraints (9) implies that the delivery arrival time of batch *b* which containing job *p* must be equal or greater than the sum of the corresponding vehicle departure time and the travel time before job *p*. Formula (10) represents the calculation method of the departure time of job *p*. Constraint (11) guarantees that a job is only allowed to be allocated to a batch for distribution. Constraint (12) represents the constraints about batches and factories. Constraint (13) determines that the deliver products does not exceed the vehicle capacity limit and meet the demands of one retailer at least. Constraint (14) ensures that all retailers' demands are met. Constraint (15) ensures that a job is assigned to only one factory. Constraint (16) guarantees that all jobs in the vehicle are delivered before returning. Constraint (17) means that time and cost-related decision variables are positive.

### Processing of travel time uncertainty

During the arrangement of distribution vehicles, there are lots of uncertainties in these processes which are difficult to be described accurately by mathematical model. And it will lead to large deviation in the actual implementation of the scheduling scheme if these factors are ignored. In this section, Monte Carlo method is used to deal with these uncertainties during the journey.

Assumed that the travel time between distribution points satisfies the normal distribution in the cause of traveling. The size of uncertain factors during vehicle driving is mainly related to the actual length of delivery routes. The longer the path, the more uncertain events occur during driving, which meets *X* ~ *N*(*μ,*$\sigma^{{2}}$):

18 $$ {\text{t}}_{i,j}^{{}} \sim N({\text{t}}_{i,j}^{{}} ,\sigma_{i,j}^{2} ) $$

The formula that converts the uncertain factors during distribution process into deterministic factors through Monte Carlo simulation is as follows:

19 $$ \mathop t\limits^{ - }_{i,j} \sim \frac{1}{T}\sum\limits_{t
= 1}^{T} {f{(}t_{i,j}^{t} ,\sigma_{i,j}{)}} $$

Since the random variable satisfies normally distributed and the size of standard deviation will affect the choice of distribution scheme. The standard deviation factor *k* is designed to control the randomness as follows:

20 $$ \sigma_{i,j}^{{}} = k^{2} \cdot d_{i,j}^{{}} $$

## Proposed solution algorithm

IPDSP has been extensively investigated and proved to be NP-hard [33]. And the proposed IDPDSP-GM-UTT can be regarded as the extension of IPDSP, which considering the distributed processing factory, muti-vehicle and uncertain batch distribution situations simultaneously. It is more complicated than traditional IPDSP and undoubtedly is also NP-hard. So, a hybrid genetic algorithm with a new three-layer representation scheme is developed in this section for the proposed mathematic model. In the proposed algorithm, a two-stage heuristic mutation scheduling strategy is designed which can achieve rapid convergence at the initial iteration of algorithm and greatly improve the probability of finding the optimal solution. Then, a tabu search is used to improve the local search performance of algorithm at the end of population iteration. The steps and strategies in the algorithm are detailed described in the following sections.

### Framework of GA-2HMS&TS

**Fig. 2:**
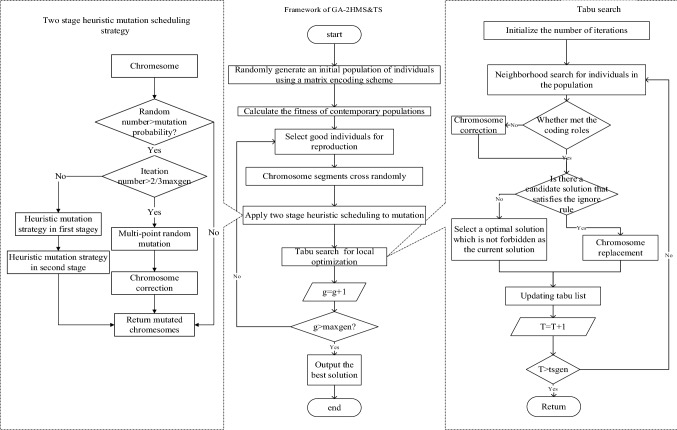
Framework of GA-2HMS&TS

The framework of GA-2HMS&TS is show in Fig. 2 and the detailed process are described in the follow sections:

#### Encoding and decoding scheme

**Fig. 3:**
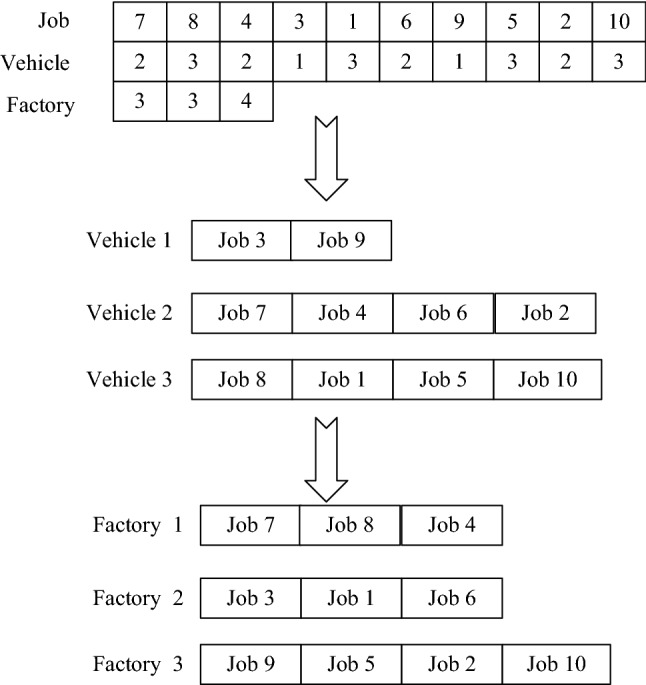
A chromosome of GA-2HMS&TS

Encoding strategy greatly affects the performance of an efficient meta-heuristic. In this paper, the IDPDSP-GM-UTT concludes jobs assignment among distributed factories, job tasks sequencing at production stage, and batch delivery of finished jobs with capacity limited vehicles at the distribution stage. A matrix of three rows is used to represent a chromosome. The *n* numbers randomly arranged in the first row show the processing sequence of *n* workpieces in a distributed hybrid flow shop. The second row represents the delivery vehicle selected by different jobs which are determined in the first row. The last row represents the production plant that are chosen by jobs. A feasible solution is shown in Fig. 3:

The detailed decoding steps are as follows:

Step 1 Calculate the production waiting time, the finish time of each job according to the job production sequence and processing workshop where the job is located. Then, determine production costs.
Step 2 Calculate the delivery waiting time of different vehicles and the delivery time according to the delivery vehicle specified by the job. Then, calculate the delivery cost.
Step 3 Calculate the cost of exceeding time window based on arrival time and time window of different jobs.
Step 4 Calculate the fixed cost of vehicles.
Step 5 Obtain the fitness value of the chromosome, matrix of production time, matrix of delivery time, and job production orders in different factories

#### Selection operation

Selection strategies are critical to population iteration, which can choose a set of better individuals for the next iteration. Binary tournaments combined with optimal individual retention strategies are used to make choices in GA-2HMS&TS. Firstly, a certain number of individuals are selected from the population at a time, and then select the best individual as one of offspring. Repeat this operation until the size of new population reaches the original size. The detailed steps are as follows:

Step 1 Determine the number to select.
Step 2 Select the chromosome with best fitness as one of offspring.
Step 3 Repeat Step 2 until the number of descendants reach population size.
Step 4 Determine whether the individual with the best fitness value exists in *NewChrome*, if it exists return *NewChrome*, otherwise put the best individual into *NewChrome.*
Step 5 Return the population *NewChrom.*

#### Crossover operation

In this paper, chromosomes are divided into two segments and crossed separately under the premise of three-layer coding rules, both of two segments adopt a two-point crossover method. The first segment represents the job order while the second segment implies the sequence of delivery vehicle. The crossover probability of two segments is *p*_*1*_ and *p*_*2*_ respectively_*:*_

21 $$ p_{1} = \frac{C}{C + V},p_{2} = \frac{V}{C + V} $$

The notation *C* represents the number of distributed factories and notation *V* represents the number of vehicles used in a single integrated scheduling.

The specific crossover procedure is as follows:

Step 1 Generate a random number *q* between 0–1.
Step 2 If *q* is greater than *p*_*1*_, the crossover process takes place in the second segment; otherwise, the first segment is crossed.
Step 3 Complete the exchange operation and return the population.

### Two-stage heuristic variation scheduling strategy

At the initial iteration of population, heuristic variation scheduling strategy for the first stage ensures that the mutant chromosome is superior than that is not mutated. While the strategy in the distribution stage guarantees that the shipping order is consistent with the finished time of jobs. In addition, the mutation strategy divides the orders completed within the same time interval into the same batch, which reduces both of the delivery time and costs in the distribution stage. Consequently, the two-stage heuristic mutation scheduling strategy can achieve rapid convergence at the initial iteration of algorithm and greatly improve the probability of finding the optimal solution. And at the end of population iteration, tabu search is used to improve the local search performance of algorithm.

The crossover probability *P*_*c*_ and mutation probability *P*_*m*_ play a vital role in the convergence of algorithm and the quality of the solution. Excessive selection of *P*_*c*_ will destroy the balance of the group and result in the destruction of good individuals, while reduces the evolution speed of population if *P*_*c*_ is too small.

Similarly, the evolution direction of the group is changeable when the selected *P*_*m*_ value is too large, while it is not conducive to the generation of new individuals. And the algorithm is prone to prematurely fall into the local optimum if *P*_*m*_ value is too small. Therefore, it is difficult to solve the integrated scheduling problem in distributed group manufacturing with a constant cross-mutation probability. In response to this problem, this article adopts a dynamic crossover probability crossover and mutation probabilities, the specific calculation formula is as follows.

22 $$ P_{c} = \left\{ {\begin{array}{*{20}c} {{\text{k}}_{1} - (P_{{{\text{c1}}}} - P_{{{\text{c2}}}} )\left( {\frac{g}{3G} + \frac{{f{ - }f_{av} }}{{3(f_{\max } { - }f_{av} )}}} \right),} & {f \ge f_{av} } \\ {0.8,} & {f < f_{av} } \\ \end{array} } \right. $$

23 $$ P_{{\text{m}}} = \left\{ {\begin{array}{*{20}c} {{\text{k}}_{{2}} + (P_{{{\text{m1}}}} - P_{{{\text{m2}}}} )\Bigg(\frac{g}{3G} + \frac{{f{ - }f_{av} }}{{3(f_{\max } { - }f_{av} )}}\Bigg),} & {f \ge f_{av} } \\ {0.2,} & {f < f_{av} } \\ \end{array} } \right. $$

where the notation *g* represents current iteration number, *G* represents the maximum number of iterations. The parameter *f* means current fitness value, the parameter *f*_*av*_ means average fitness values to date and the parameter *f*_*max*_ means maximum fitness values so far. The parameter *k*_*1*_ represents the cross cardinality and *k*_*2*_ represents the mutation cardinality.

#### Heuristic mutation scheduling strategy in first stage

In the first-stage, chromosome segment is revised according the location of retailers and factories. The first-stage segment is revised to a better segment which has least procession time during production. The detailed operation method is as follows:

Step 1 Obtain the chromosomes that need to be muted.
Step 2 Determine the factory which job *p* is processed according to the encoding strategy of chromosomes.
Step 3 Calculate the process finishing time of each job according to processing time of job *p* in each factory.
Step 4 Calculate the processing duration of each factory.
Step 5 Taking the job *p* with shortest processing time from factory with longest processing time and placed job *p* into factory *f* with shortest processing duration.
Step 6 Calculate the chromosome fitness value after adjustment and recording the chromosome and fitness value after each adjustment.
Step 7 Repeat *Step* *6* until the number of iterations is reached.
Step 8 End the cycle and return the chromosome after first-stage heuristic mutation.

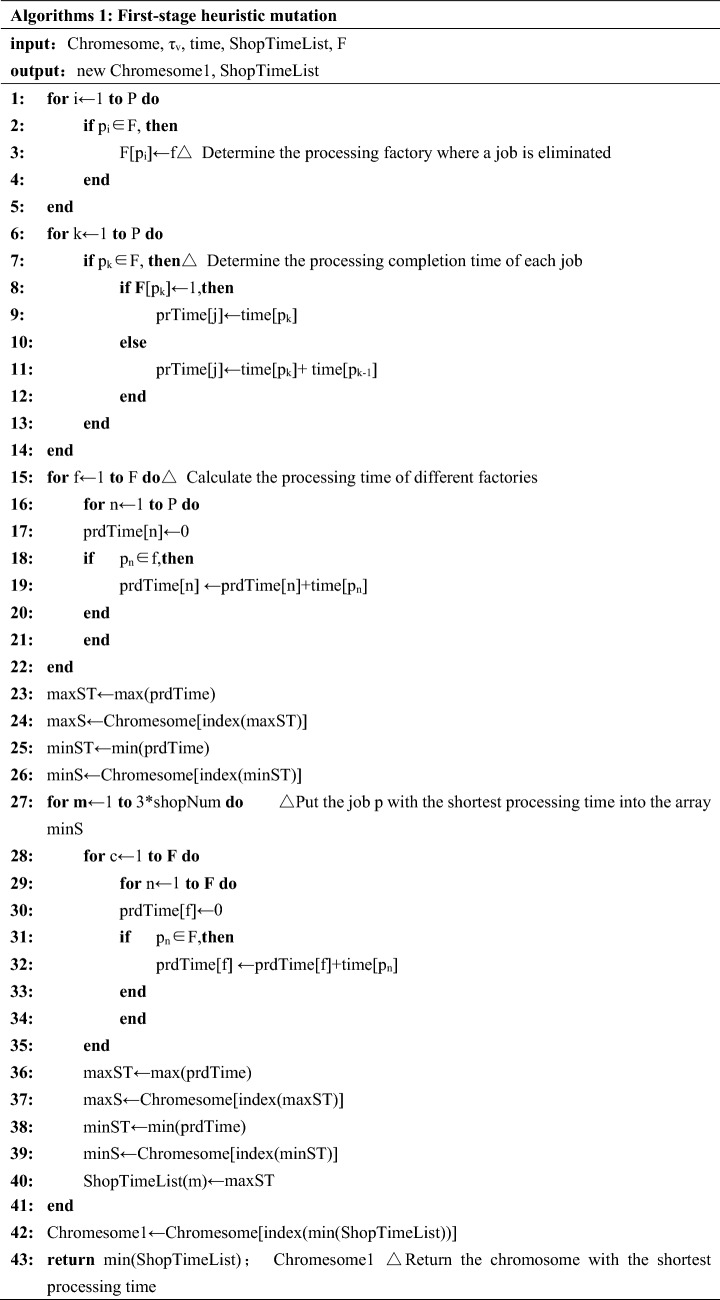


#### Heuristic mutation scheduling strategy in second stage

In the second-stage, divide jobs *p* into different batches according to the vehicle capacity limit *τv* and product completion time. Carry out the second segment coding mutation on chromosomes which obtained from Sect. Encoding and decoding scheme according to the principle of idle vehicle priority. The detailed operation method is as follows:
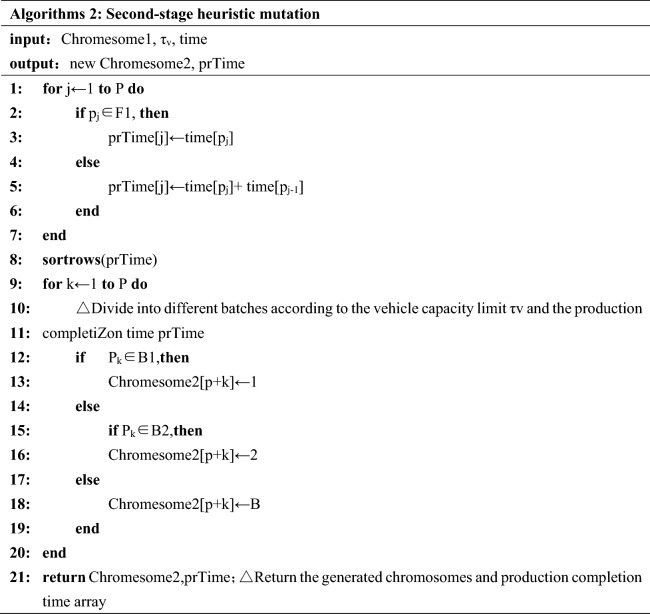


### Tabu search algorithm for local optimization

The length of tabu length plays an important role in the execution of tabu search algorithm. If it is too long, it will lead to the less efficiency of the algorithm. On the contrary, if it is too short, it will lead to the algorithm falls into local optimum. Therefore, this paper sets dynamically tabu length according to job number and iteration number. The specific value of the length is as flows:

24 $$ ({\text{Tabulen}}) = \left\{ {\begin{array}{*{20}c} {4p \times \frac{k}{{{\text{stop}}L}},} & {{\text{0 < k}} \le \frac{1}{2} \times {\text{stop}}L} \\ {p \times \frac{k}{{{\text{stop}}L}},} & {\frac{1}{2} \times {\text{stop}}L{\text{ < k}} \le {\text{stop}}L} \\ \end{array} } \right. $$

where Tabulen denotes the length of tabu table, *k* represents the number of iterations and stop*L* means the maximum iteration *z*

The specific steps of tabu search are as follows:

Step 1 Select initial individuals.

Randomly select individuals after cross and mutation for taboo operations and empty the tabu table *T*.

Step 2 Neighborhood search.

Legality adjustment to realize neighborhood search operation.

Step 3 Identify candidate solution.

Select *bestS* from the candidate solutions that satisfy the rule.

Step 4 Neighborhood evaluation.

Calculate the fitness value of the candidate solution and compare the current solution fitness value fit(Si) with the candidate solution fitness value fit(si'). If the fitness value of candidate solution is better than the current solution, replace *Si* with *Si'* and update the tabu list *T* if *Si'* dos not exists in tabu list.

Step 5 Determine whether the termination condition is met.

If the termination condition of the algorithm is satisfied, return the result of local search. Otherwise, repeat Step 2.

## Experimental results and discussions

### Experimental design

All algorithms involved in this article are developed in MATLAB R2016a and run on a computer with Intel Core^tm^ i5-4200H 3.40 GHz CPU and 8 GB RAM under Microsoft Windows 10 environment. Since the IDPDSP-GM-UTT is a new combinatorial scheduling problem and no benchmark instances have been found in the literature, we set up two sets of test problems randomly. The parameters involved in the test example are shown in Table 1.

**Table 1:** Related parameters

Parameters	Values
Processing time of job *p* at stage *k* in factory *f*	Unidrnd (1,10)
Unit processing cost of job *p* in factory *f*	Unidrnd (1,10)
Distance from factory *f* to retailer *i*	Unidrnd (10,30)
Distance from retailer *i* to retailer *j*	Unidrnd (10,30)
Variable transportation cost of vehicle *v* per unit time	4
Unit tardiness cost of job *p* for retailer *i*	Unidrnd (1,10)
Unit earliness cost of job *p* for retailer *i*	Unidrnd (1,10)
Time window of job *p*	[T-Unidrnd (0, 50), *T* + Unidrnd (0, 50)] $T = \sum\limits_{{{\text{p}} = 1}}^{P} {\sum\limits_{f = 1}^{F} {\sum\limits_{t = 1}^{T} {\upsilon (\pi_{f}^{{\text{p}}} ,t)} } }$
Fixed transportation cost of vehicle *v*	2
Fixed processing cost of job *p*	3

To verify the effectiveness of GA-2HMS&TS, three representative heuristic algorithms in the existing literature for solving IPDSP, i.e., minimal critical ratio (CR) [34], minimal slack (MS) [35], and earliest due date heuristics (EDD) [36], are introduced for comparison. Besides, to prove the effectiveness of the proposed improvement strategy, GA-TS without heuristic mutation scheduling strategy and standard GA are also used to compare with GA-2HMS&TS to solve the proposed problem. In order to further compare the performance of the algorithms, one performances metric, relative error (RE), is adopted to evaluate the convergence and distribution of solutions sets. RE is widely used for benchmark comparison and has been proved that it can effectively compare the stability of heuristic algorithms. The calculation formula of RE is as follows:

25 $$ {\text{RE}} = \frac{{C_{i} - C_{\min } }}{{C_{\min } }} $$

where *C*_min_ means the minimum cost which is calculated by the Eq. (1) among six compared algorithms. *C*_*i*_ expresses the total cost value obtained by one specific algorithm.

There are four parameters that are vitally important in GA-2HMS&TS, namely population size (NIND), maximum iteration (Max), crossover rate (*P*_*c*_), mutation rate (*P*_*m*_). And each parameter has four level. The detailed parameter levels are shown in Table 2. To find the optimal parameters, several experiments are carried out with diverse parameter settings and obtained results are analyzed using Taguchi approach. Based on the orthogonal array L_*16*_(4^4^), different parameter combinations(3 × 40 × 15) are shown in Table 3.

**Table 2:** Factors and their levels for GA-2HMS&TS

Instance	NIND	Max	*P*_c_	*P*_m_
1	50	200	0.6	0.05
2	100	300	0.7	0.1
3	150	400	0.8	0.15
4	200	500	0.9	0.2

The GA-2HMS&TS with each parameter combinations is performed 20 times independently and the average relative error (ARE) over 20 independent results are shown in Table 3. The significance rank of four parameters is exhibited in Table 4. Based on the aforementioned experimental results from Table 4, it can be seen that *P*_*c*_ and NIIND play the most important and second roles. A reasonable parameter combination is suggested as follows: population size NIND = 200, Maximum iteration Max = 400, initial crossover probability *P*_*c*_ = 0.8, initial mutation probability *P*_*m*=_ 0.2. self-adapting crossover probability *P*_*c1*_ = 0.9 and *P*_*c2*_ = 0.6, self-adapting mutation probability *P*_*m1*_ = 0.2 and *P*_*m2*_ = 0.1, *k1* = 0.9, *k2* = 0.1.

**Table 3:** Orthogonal experiments and response values of GA-2HMS&TS

Trail	Parameter				ARE
	NIND	Max	*P*_*c*_	*P*_*m*_	
1	50	200	0.6	0.05	3.086
2	50	300	0.7	0.1	2.534
3	50	400	0.8	0.15	2.183
4	50	500	0.9	0.2	2.375
5	100	200	0.7	0.15	2.699
6	100	300	0.6	0.2	2.47
7	100	400	0.9	0.05	2.408
8	100	500	0.8	0.1	2.366
9	150	200	0.8	0.2	2.153
10	150	300	0.9	0.15	2.792
11	150	400	0.6	0.1	2.513
12	150	500	0.7	0.05	2.588
13	200	200	0.9	0.1	2.114
14	200	300	0.8	0.05	1.904
15	200	400	0.7	0.2	1.736
16	200	500	0.6	0.15	2.567

### Experimental results

The combination of parameter *f*, *p*, *k* is used to describe a set of experimental instances. For example, 3 × 10 × 5 represents an instance with 3 factories, 10 jobs and 5 stages. The experimental results of six algorithms for two set of examples with different size are shown in Tables 5 and 6, respectively.

**Table 4:** Response and rank of parameters for the GA-2HMS&TS

Instance	NIND	Max	*P*_*c*_	*P*_*m*_
1	2.5445	2.513	2.659	2.4965
2	2.48575	2.425	2.38925	2.38175
3	2.5115	2.21	2.1515	2.56025
4	2.08025	2.474	2.42225	2.1835
Delta	0.46425	0.303	0.5075	0.37675
Rank	2	4	1	3

**Table 5:** Results with different algorithms for small sized examples

Instance	*f* × *p* × *k*	MS	EDD	CR	GA	GA-TS	GA-2HMS&TS
1	2 × 10 × 5	35.08	35.06	34.32	29.28	28.89	28.35
2	2 × 10 × 10	43.54	43.33	43.11	36.47	34.81	34.69
3	2 × 20 × 5	78.29	77.26	77.75	62.44	60.81	60.81
4	2 × 20 × 10	98.88	97.38	95.33	77.20	72.16	71.38
5	3 × 10x5	47.01	47.58	48.66	38.19	36.14	36.08
6	3 × 10 × 10	55.75	55.12	53.40	42.54	41.46	41.29
7	3 × 20 × 5	108.52	106.93	103.40	87.92	81.26	81.26
8	3 × 20 × 10	127.82	125.46	125.98	100.86	93.23	92.11
9	4 × 10 × 5	70.18	68.85	66.89	54.96	53.51	53.23
10	4 × 10 × 10	75.39	75.03	72.60	58.13	56.35	55.74
11	4 × 20 × 5	173.10	165.15	158.62	130.78	123.93	123.44
12	4 × 20 × 10	198.64	195.53	183.26	151.62	139.75	138.49
13	5 × 10 × 5	84.13	86.19	83.59	65.55	62.87	62.17
14	5 × 10 × 10	100.13	97.40	93.89	72.33	70.99	69.35
15	5 × 20 × 5	216.44	214.02	210.90	158.46	152.58	149.84
16	5 × 20 × 10	236.29	232.67	231.11	175.44	164.68	161.59

**Fig. 4:**
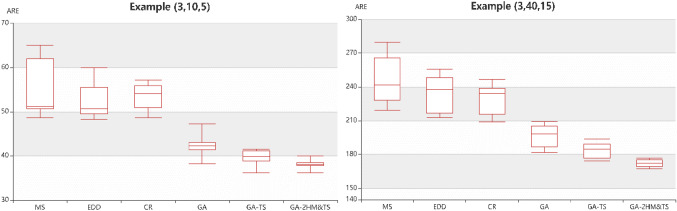
Boxplot graphs of six algorithms

Furthermore, Fig. 4 shows the comparison boxplot graphs of six algorithms for the two selected problem (3 × 10 × 5, and 3 × 40 × 15) respectively. It can be seen that the optimal value obtained by the GA-2HMS&TS is better than that of the other algorithms and the range of fluctuations is also much smaller. Different from the GA, the GA-2HMS&TS optimizes the fitness value of chromosomes in the process of variation. In addition, the chromosomes are locally optimized after mutation by tabu search algorithm, which ensures that chromosomes evolve not in a bad direction. And we can summarize that the GA-2HMS&TS in this work is superior than GA and GA-TS.

Since three heuristics (GA, GA-TS, GA-2HMS&TS) are much faster than MS, EDD and CR. The ARE, the best value (BRE), and the worst value (WRE) of RE are applied to compare these three heuristics in detail. The calculation results are shown in Tables 7 and 8. It can be observed that three meta-heuristic algorithms (GA, GA-TS, GA-2HMS&TS) can get better results than three heuristic algorithms. And the CR has better RE than MS and EDD, which indicates that the CR is more suitable for the IDPDSP-GM-UTT. And among all the three meta-heuristic algorithms, GA-2HMS&TS can get the best results for both large-scale and small-scale problems.

**Table 6:** Results with different algorithms for large sized examples

Instance	*f* × *p* × *k*	MS	EDD	CR	GA	GA-TS	GA-2HMS&TS
1	2 × 40 × 15	212.69	209.28	207.92	173.08	172.34	166.74
2	2 × 40 × 20	286.41	274.23	268.04	220.73	224.09	213.66
3	2 × 50 × 15	250.55	240.37	245.16	205.78	199.52	191.35
4	2 × 50 × 20	351.77	340.42	321.49	258.44	254.77	246.7
5	3 × 40 × 15	219.01	212.72	208.96	181.80	174.30	167.34
6	3 × 40 × 20	288.78	296.53	282.88	243.90	233.54	225.19
7	3 × 50 × 15	304.40	287.31	278.57	221.47	210.48	210.48
8	3 × 50 × 20	354.98	346.12	314.98	269.20	254.19	242.57
9	4 × 40 × 15	257.87	240.94	248.30	194.00	189.04	185.24
10	4 × 40 × 20	298.15	292.08	282.13	227.24	214.10	206.96
11	4 × 50 × 15	341.46	333.97	325.85	274.45	260.17	255.37
12	4 × 50 × 20	391.80	363.81	363.28	300.55	278.93	268.28
13	5 × 40 × 15	272.88	283.79	264.00	210.57	201.91	198.11
14	5 × 40 × 20	328.99	309.24	296.62	231.51	225.80	216.51
15	5 × 50 × 15	375.71	381.34	378.23	297.62	283.58	274.84
16	5 × 50 × 20	454.85	443.97	426.45	324.82	305.81	291.53

**Table 7:** RE value of different algorithms for small sized examples

Instance	*f* × *p* × *k*	MS	EDD	CR	GA			GA-TS			GA-2HMS&TS		
		RE	RE	RE	BRE	ARE	WRE	BRE	ARE	WRE	BRE	ARE	WRE
1	2 × 10 × 5	23.74	23.68	21.07	3.29	5.91	9.79	1.91	2.06	6.75	0.00	0.81	1.36
2	2 × 10 × 10	25.51	24.92	24.27	5.13	7.21	12.94	0.36	2.85	7.30	0.00	1.14	1.86
3	2 × 20 × 5	28.75	27.05	27.86	2.68	6.11	14.17	0.00	3.71	8.69	0.00	1.48	3.22
4	2 × 20 × 10	38.52	36.43	33.55	8.15	9.97	11.89	1.09	3.21	11.96	0.00	1.28	4.14
5	3 × 10 × 5	30.29	31.87	34.88	5.84	6.48	9.48	0.18	2.44	7.33	0.00	1.01	2.14
6	3 × 10 × 10	35.02	33.49	29.33	3.02	4.98	15.76	0.40	4.49	8.92	0.00	1.86	3.43
7	3 × 20 × 5	33.55	31.59	27.24	8.20	9.81	10.59	0.00	2.07	10.34	0.00	1.28	3.91
8	3 × 20 × 10	38.77	36.21	36.77	9.50	9.33	11.69	1.22	3.64	8.09	0.00	1.3	1.75
9	4 × 10 × 5	31.84	29.34	25.66	3.25	6.61	13.46	0.52	3.09	9.75	0.00	1.21	2.41
10	4 × 10 × 10	35.26	34.6	30.25	4.29	8.95	13.00	1.09	4.20	10.53	0.00	2.04	3.67
11	4 × 20 × 5	40.23	33.79	28.50	5.95	10.47	17.20	0.40	4.96	10.62	0.00	1.43	3.2
12	4 × 20 × 10	43.43	41.19	32.33	9.48	13.78	18.41	0.91	6.75	11.29	0.00	2.04	4.27
13	5 × 10 × 5	35.33	38.63	34.46	5.44	8.29	17.27	1.13	4.49	9.79	0.00	1.22	3.06
14	5 × 10 × 10	44.39	40.44	35.38	4.29	8.91	20.81	2.37	5.88	9.08	0.00	1.43	3.31
15	5 × 20 × 5	44.45	42.83	40.75	5.75	10.29	16.69	1.83	6.73	11.58	0.00	1.07	3.09
16	5 × 20 × 10	46.23	43.99	43.02	8.57	12.07	20.20	1.91	6.61	11.44	0.00	1.89	4.21
Average		35.96	34.38	31.58	5.80	8.69	14.58	0.95	4.19	9.59	0.00	1.41	3.06

**Fig. 5:**
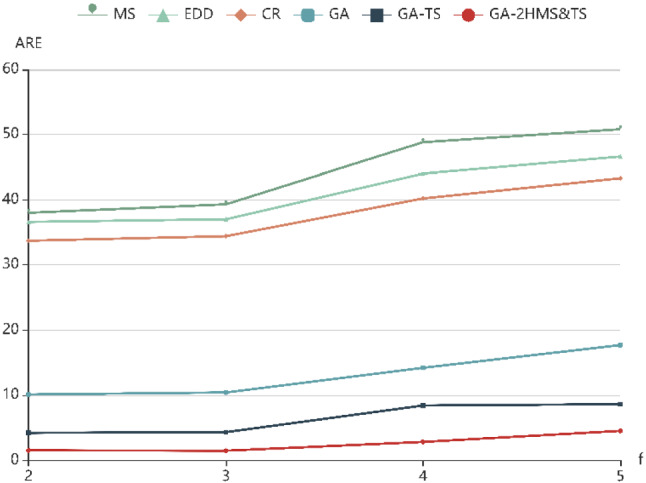
ARE with different numbers of factories (f)

**Fig. 6:**
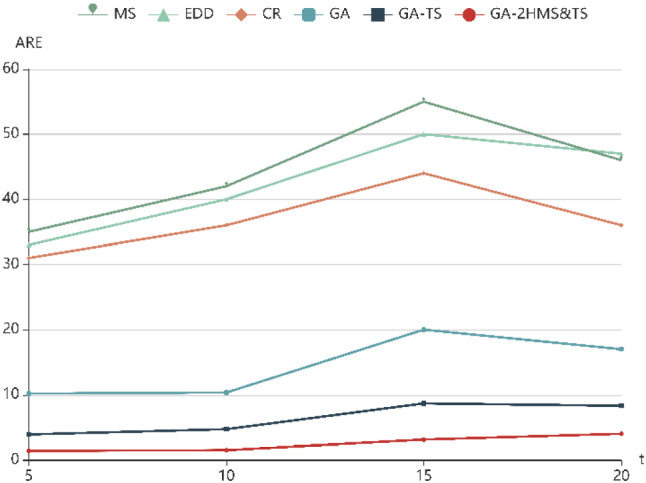
ARE with different stages in a factory (t)

**Fig. 7:**
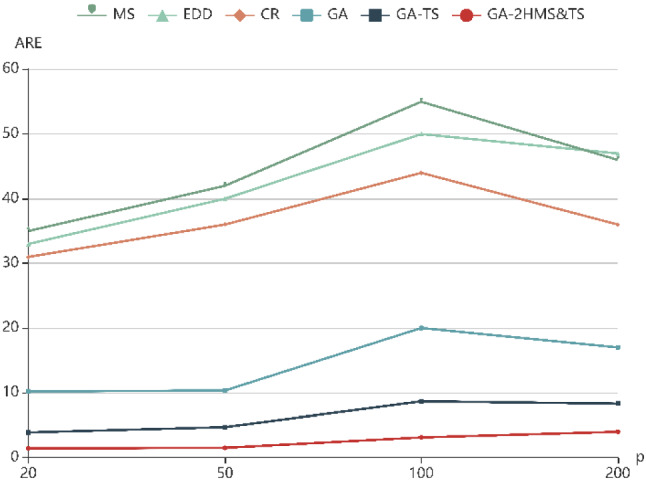
ARE with different numbers of jobs (p)

To further evaluate the performance of six algorithms with the change of parameter values, ARE with different parameters (*f*, *t*, *p*) are shown in Fig. 5, 6 and 7, respectively. It can be seen that the ARE of GA-2HMS&TS obviously are better than those obtained by GA and GA-TS. On the other hand, it can be observed that the there is an upward trend with the increase of four parameters, which indicates that the ARE increases with the increase of example size.

It can be seen that the GA-2HMS&TS has strong search ability in both exploration and exploitation phases for solving the proposed model. In the GA-2HMS&TS, a two-stage heuristic mutation scheduling strategy is adopted to enhance the search ability. Additionally, the tabu search approach is employed to optimize mutant individuals, which can strengthen the local search ability of GA-2HMS&TS. Therefore, the proposed algorithm has better performance than others for handling the proposed model.

### Parameter sensitivity analysis

**Fig. 8:**
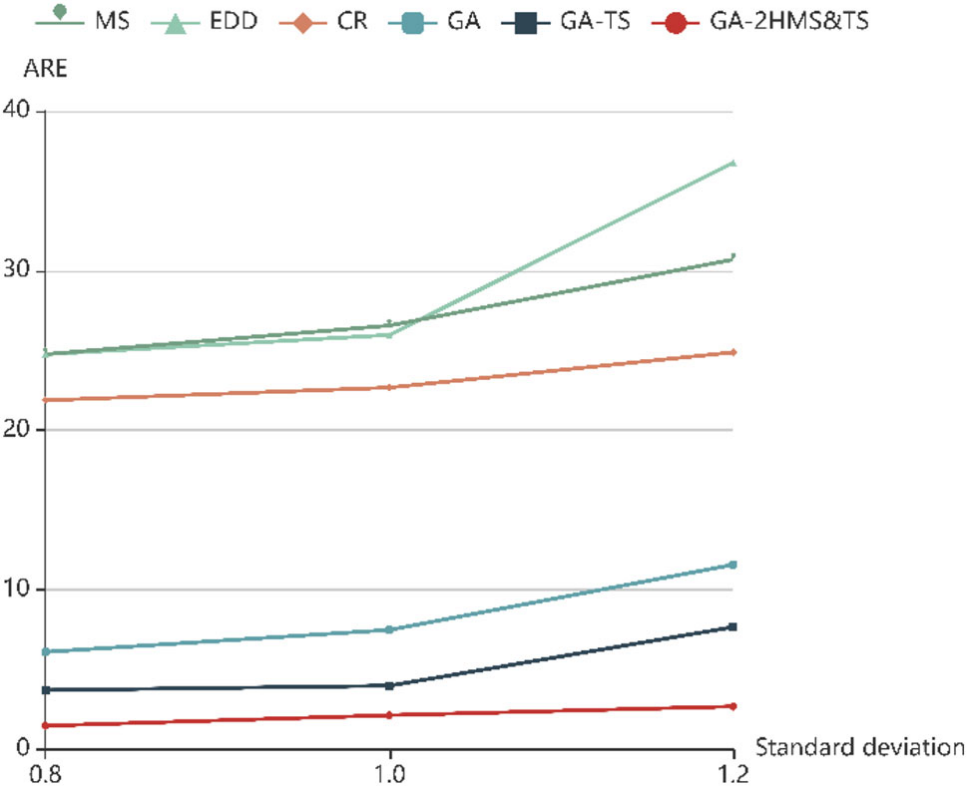
ARE of different algorithms for different road conditions

To verify the robustness of the proposed model to deal with the uncertainty of travel time, the Monte Carlo method mentioned in Sect. Processing of travel time uncertainty is introduced to handle uncertain factors during the journey. Since the choice of standard deviation factor will affects the randomness, the standard deviation factor *k* is set as 0.8, 1.0 and 1.2, respectively. The RE values of the six algorithms under different road conditions are shown in Table 9 and Fig. 8.

**Table 8:** RE value of different algorithms for large sized examples

Instance	*f* × *p* × *k*	MS	EDD	CR	GA			GA-TS			GA-2HMS&TS		
		RE	RE	RE	BRE	ARE	WRE	BRE	ARE	WRE	BRE	ARE	WRE
1	2 × 40 × 15	27.56	25.51	24.70	3.80	6.10	23.32	3.80	4.32	10.72	0.00	2.85	5.33
2	2 × 40 × 20	34.05	28.35	25.45	3.31	14.63	28.91	3.31	6.02	22.29	0.00	3.57	8.95
3	2 × 50 × 15	30.94	25.62	28.12	7.54	8.00	20.34	7.54	5.26	14.37	0.00	2.38	9.19
4	2 × 50 × 20	42.55	37.95	30.28	4.73	8.50	23.70	4.73	7.05	17.20	0.00	4.36	11.89
5	3 × 40 × 15	30.88	27.12	24.87	8.64	9.27	30.09	8.64	10.62	29.80	0.00	3.81	8.55
6	3 × 40 × 20	28.24	31.68	25.62	8.31	10.30	23.60	8.31	11.59	27.46	0.00	5.73	11.55
7	3 × 50 × 15	44.62	36.50	32.35	5.22	7.88	20.04	5.22	6.83	25.89	0.00	3.89	9.3
8	3 × 50 × 20	46.34	42.69	29.85	10.98	12.61	23.88	10.98	11.43	30.56	0.00	2.72	10.54
9	4 × 40 × 15	39.21	30.07	34.04	4.73	9.53	23.36	4.73	10.40	25.20	0.00	3.76	8.44
10	4 × 40 × 20	44.06	41.13	36.32	9.80	14.09	21.60	9.80	11.72	20.36	0.00	2.44	10.32
11	4 × 50 × 15	33.71	30.78	27.60	7.47	10.37	25.04	7.47	6.92	21.78	0.00	3.61	6.79
12	4 × 50 × 20	46.04	35.61	35.41	12.03	18.59	25.75	12.03	9.52	27.67	0.00	4.17	9.74
13	5 × 40 × 15	37.74	43.25	33.26	6.29	7.90	22.41	6.29	7.98	16.62	0.00	2.34	6.27
14	5 × 40 × 20	51.95	42.83	37.00	6.93	14.48	24.91	6.93	9.26	21.05	0.00	4.06	8.18
15	5 × 50 × 15	36.70	38.75	37.62	8.29	11.92	30.62	8.29	8.29	27.26	0.00	3.58	9.54
16	5 × 50 × 20	56.02	52.29	46.28	11.42	21.03	38.77	11.42	10.40	33.25	0.00	6.27	12.61
Average		39.41	35.63	31.80	7.46	11.58	25.4	3.37	8.60	23.22	0.00	3.72	9.20

**Fig. 9:**
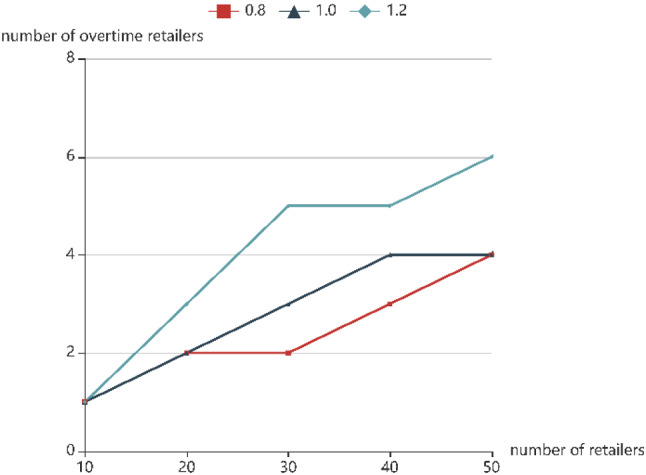
The number of retailers that violate time window constraints

To analyze the sensitivity of the number of retailers, experimental analysis under three different road conditions is made respectively, which is shown in Fig. 9.

With the increase of the number of service retailers, the number of retailers exceeding the time window shows a significant increase trend for all of three conditions. When serving the same number of retailers, the greater the uncertainty of travel time, the more retailers violate the service time window. In addition, we find that GA-2HMS&TS has better performance than the other algorithms from Fig. 8 although the uncertainty of the journey has increased.

It can be found from the above results that the proposed method for solving the uncertain factors is effective. The uncertain factors during the distribution stage ought not to be overlook, especially when some unexpected conditions happened, such as over numerate received orders, bad weather or serious road conditions. Decision makers need to appropriately increase the number of distribution vehicles to reduce the influence of uncertainty disturbance on enterprise operation to avoid unnecessary losses.

What’s more, to verify the validity of the model and algorithm for different distributions, we did sensitivity analysis with Normal, Poisson and Uniform distribution. The RE values of the six algorithms under different distribution conditions with instance 3f10p5k are shown in Table 10.

**Table 9:** RE of different algorithms for different road conditions

k	MS	EDD	CR	GA			GA-TS			GA-2HMS&TS		
	RE	RE	RE	BRE	ARE	WRE	BRE	ARE	WRE	BRE	ARE	WRE
0.8	24.75	24.75	21.86	0.48	6.11	14.17	0.00	3.71	8.69	0.00	1.48	3.22
1.0	26.55	25.96	22.66	0.44	7.49	14.85	1.44	3.99	9.46	0.00	2.14	3.96
1.2	30.69	36.77	24.87	0.76	11.58	22.37	3.54	7.67	10.22	0.00	2.69	3.88

**Table 10:** RE of different algorithms for different distribution conditions

Distribution	MS	EDD	CR	GA			GA-TS			GA-2HMS&TS		
	RE	RE	RE	BRE	ARE	WRE	BRE	ARE	WRE	BRE	ARE	WRE
Normal	24.75	24.75	21.86	0.48	6.11	14.17	0.00	3.71	8.69	0.00	1.48	3.22
Poisson	21.14	22.77	28.37	1.69	6.83	16.14	0.36	4.48	9.65	0.00	1.49	4.94
Uniform	25.49	26.91	31.06	0.67	7.38	15.64	1.54	4.16	11.14	0.00	2.17	5.72

It can be seen from Table 10 that the GA-2HMS&TS can still obtain the best RE value under the assumption of different distributions. The fluctuation range of experimental results under different distributions is also small, which proves that the designed model and algorithm are still valued under the assumption where the travel time meets Poisson and Uniform distribution.

## Conclusion

This paper proposes a new IDPDSP-GM-UTT, in which products are firstly processed in distributed hybrid flow shops and subsequently delivered to several customers in batches with capacity limited vehicles. An integrated scheduling model is established considering uncertain travel time to minimize the total costs. To solve the IDPDSP-GM-UTT, an improved genetic algorithm with two-stage heuristic mutation scheduling strategy and tabu search is designed. To assess the superiority of GA-2HMS&TS in solving the considered scheduling problem, several experiments by adopting test examples with different scales are performed. Three fast heuristics (CR, EDD, and MS) and two baseline algorithms (GA and GA-TS) are chosen for comparisons. The obtained comparative results exhibit that the GA-2HMS&TS has significantly superiority for solving the IDPDSP-GM-UTT. Moreover, the sensitivity analysis indicates that the proposed method for solving the uncertain factors is effective and the uncertain factors during the distribution stage ought not to be overlook.

The future research is to extend the model to include more uncertain factors in the process of production and distribution, such as uncertain factors of job demands or cost. This could likely lead to the constructed model much more complicated but would yield results that are more closely aligned with practice.
